# Prognostic Impact of the Symptom of New-Onset Atrial Fibrillation in Acute Myocardial Infarction: Insights From the NOAFCAMI-SH Registry

**DOI:** 10.3389/fcvm.2021.677695

**Published:** 2021-09-22

**Authors:** Jiachen Luo, Baoxin Liu, Hongqiang Li, Siling Xu, Mengmeng Gong, Zhiqiang Li, Xiaoming Qin, Beibei Shi, Chuanzhen Hao, Ji Zhang, Yidong Wei

**Affiliations:** Department of Cardiology, Shanghai Tenth People's Hospital, Tongji University School of Medicine, Shanghai, China

**Keywords:** acute myocardial infarction, atrial fibrillation, symptom, mortality, heart failure, ischemic stroke

## Abstract

**Background:** New-onset atrial fibrillation (NOAF) is a common complication during acute myocardial infarction (AMI) and sometimes can be completely asymptomatic, but the clinical implications of these asymptomatic episodes require further characterization. The objective of this study was to investigate the short- and long-term prognostic impact of post-MI NOAF based on the presence of AF-related symptoms.

**Methods:** The New-Onset Atrial Fibrillation Complicating Acute Myocardial Infarction in ShangHai (NOAFCAMI-SH) registry was a retrospective cohort including participants with AMI without a documented history of AF. Patients with NOAF were divided into two groups according to the AF-related symptoms. The primary endpoint was all-cause mortality.

**Results:** Of 2,399 patients included, 278 (11.6%) developed NOAF of whom 145 (6.0%) with asymptomatic episodes and 133 (5.5%) with symptomatic ones. During hospitalization, 148 patients died [106, 10, and 32 in the sinus rhythm (SR), asymptomatic, and symptomatic NOAF groups, respectively]. After multivariable adjustment, only symptomatic NOAF was associated with in-hospital mortality [odds ratio (OR): 2.32, 95% confidence interval (CI): 1.36–3.94] compared with SR. Over a median follow-up of 2.7 years, all-cause mortality was 3.2, 12.4, and 11.8% per year in the SR, asymptomatic, and symptomatic NOAF groups, respectively. After adjustment for confounders, it was the asymptomatic NOAF [hazard ratio (HR): 1.61, 95% CI: 1.09–2.37) rather than the symptomatic one (HR: 1.37, 95% CI: 0.88–2.12) that was significantly related to mortality. Similar results were also observed for cardiovascular mortality [HRs and 95% CI were 1.71 (1.10–2.67) and 1.25 (0.74–2.11) for asymptomatic and symptomatic NOAF, respectively]. Both asymptomatic and symptomatic NOAF episodes were associated with heart failure, whereas only those with symptomatic NOAF were at heightened risk of ischemic stroke. Our exploratory analysis further identified patients with asymptomatic high-burden NOAF as the highest-risk population (mortality: 19.6% per year).

**Conclusion:** Among patients with AMI, symptomatic NOAF is related to in-hospital mortality and asymptomatic NOAF is associated with poor long-term survival.

**Registration:** URL: https://clinicaltrials.gov/; Unique identifier: NCT03533543.

## Introduction

Atrial fibrillation (AF) is one of the most common arrhythmias worldwide, with a growing public burden due to the aging of the population. Atrial fibrillation is often intermittent and asymptomatic; sometimes it can only be detected during the diagnostic evaluation of patients presenting with cryptogenic stroke (1, 2). Debates over the screening modality, prognostic impact, and management of these asymptomatic AF episodes still exist (3–10).

Among patients with acute myocardial infarction (AMI), nearly 5–20% of whom will develop new-onset atrial fibrillation (NOAF), which is generally accompanied by increased risks of subsequent death and ischemic stroke (11, 12). Similar to the condition in the general population, NOAF during AMI can also be completely asymptomatic (13). Given the potential adverse impact of asymptomatic AF, researches focusing on the prevalence, clinical profiles, as well as prognostic implications of asymptomatic NOAF complicating AMI are of great clinical importance in helping the decision-making for out-patient ECG monitoring strategy as well as stroke and decompensated heart failure (HF) prophylaxis (14). However, until now, only in the sensitivity analysis of an observational AMI registry had researchers explored the impact of asymptomatic AF on prognosis (15).

Accordingly, using data from the New-Onset Atrial Fibrillation Complicating Acute Myocardial Infarction in ShangHai (NOAFCAMI-SH) registry, we aimed to perform a retrospective analysis to describe the clinical features and to investigate the impact of asymptomatic and symptomatic NOAF during AMI on in-hospital and long-term survival.

## Methods

### Study Population

The design of the NOAFCAMI-SH registry has been previously described (16, 17). In brief, this is a retrospective cohort study from a tertiary academic medical center, which included patients who experienced an AMI, did not have a medical history of AF, and received continuous electronic monitoring (CEM) during hospitalization between February 2014 and March 2018. For the present analysis, all NOAFCAMI-SH participants were included, while event-free survival was only analyzed among individuals who were discharged alive with morbidity follow-up available. Supplementary Figure 1 illustrates a CONSORT diagram of the study population. This study was conducted according to the Declaration of Helsinki, and the protocol of the NOAFCAMI-SH registry had been approved by the ethics committee of the Shanghai Tenth People's Hospital. Informed consent was not required as all data were deidentified during the analytic stages.

### Asymptomatic and Symptomatic NOAF Ascertainment

The occurrence of AF episodes was identified according to the individuals' CEM data. AF was diagnosed based on the consensus guidelines as follows: absolutely irregular RR intervals, no distinct P waves, and lasted for at least 30 s (14). NOAF was defined as patients without a history of AF who developed the first documented AF during the index AMI hospitalization. Patients would be systematically interviewed for their symptoms whenever an AF episode presented. Symptomatic NOAF was determined if the occurrence of NOAF event was simultaneously accompanied by any discomfort (e.g., chest tightness, palpitation, shortness of breath, etc.) or the need for emergent cardioversion. Asymptomatic NOAF was determined as any asymptomatic events of NOAF (13). The analyzed population was divided into three groups: sinus rhythm (SR), asymptomatic NOAF, and symptomatic NOAF.

### Baseline Covariates

Baseline covariates consisted of patient demographics, medical history, in-hospital examination, and medications, which were ascertained by a detailed review of electronic medical records during or before the index hospitalization. Demographics included age, sex, smoking status, and body mass index. Medical history included hypertension, diabetes, hyperlipidemia, chronic kidney disease (CKD), HF, MI, percutaneous coronary intervention (PCI), peripheral artery disease (PAD), and stroke/transient ischemic attack (TIA). The in-hospital examination included creatinine, peak-TnT, peak NT-pro BNP, and angiographic and echocardiographic data. Medications included the use of antiplatelet agents, oral anticoagulants, ACE inhibitor/angiotensin receptor blocker (ACEI/ARB), β-blocker, diuretic, and amiodarone.

### Outcome Measures

The primary outcome was all-cause death. Secondary outcomes included cardiovascular death, HF hospitalization, and ischemic stroke. All deaths without a definite non-cardiovascular cause (e.g., severe pneumonia, malignant tumors, end-stage renal disease, traffic accidents, etc.) would be treated as cardiovascular deaths. HF hospitalization was defined as any admission with a primary diagnosis of HF at discharge requiring intravenous diuretics. Ischemic stroke was defined as the occurrence of a new focal neurologic deficit considered to be ischemic in origin, with signs or symptoms lasting over 24 h. Patients were followed from the index discharge to the date of the presence of an outcome of interest, death, or last follow-up (April 2019), whichever came first. Clinical outcomes were evaluated by a comprehensive review of the patient's medical records.

### Statistical Analysis

Categorical variables were presented as frequencies and proportions and were compared with the χ^2^ or Fisher's exact test, as appropriate. Continuous variables were presented as means or medians and were compared with the one-way analysis of variance or Kruskal–Wallis test, as appropriate.

Treating SR as the reference, multivariable logistic regression models were established to investigate the association of asymptomatic and symptomatic NOAF with in-hospital death. Odds ratios (ORs) and 95% confidence intervals (CIs) were calculated using three multivariable logistic regression models. In model 1, we adjusted for age and sex. In model 2, we further adjusted for cardiovascular risk factors (current smoker, hypertension, diabetes, CKD, previous MI, previous stroke/TIA, and PAD). In model 3, we further adjusted for admission characteristics [systolic blood pressure (SBP), heart rate, initial Killip class, out-of-hospital cardiac arrest, and left ventricular ejection fraction (LVEF)] and in-hospital PCI. For long-term survival analyses, event-free survival curves were estimated with the Kaplan–Meier method and compared using log-rank tests. We calculated hazard ratios (HRs) with 95% CIs by multivariable Cox proportional hazards analyses, and the candidate covariates included the following: (i) the Global Registry Acute Coronary Events (GRACE) risk score as a whole and (ii) variables in model 3. Of note, for the ischemic stroke evaluation, the individual components (age, sex, a history of HF, hypertension, diabetes, stroke/TIA, and vascular disease) in the CHA_2_DS_2_-VASc score were adjusted. The assumption of proportional hazards was verified by a visual examination of the log (minus log) curves.

The propensity score method was also used to compare the SR with either asymptomatic or symptomatic NOAF. Binary logistic regression analysis was used to calculate propensity scores to balance baseline characteristics (covariates listed in Supplementary Material). Two sets of propensity scores were calculated, one for comparing SR with asymptomatic NOAF and the other to compare SR with symptomatic NOAF. Matching was performed with a 1:3 matching protocol without replacement, using a caliper width equal to 0.10 of the SD of the propensity score.

### Subgroup and Sensitivity Analyses

The associations between asymptomatic and symptomatic NOAF and mortality were explored in subgroups as follows: age (≥75 vs. <75 years), gender (male vs. female), AMI type (STEMI vs. NSTEMI), and whether the patient underwent PCI (yes vs. no). Additionally, several sensitivity analyses were also conducted. First, adjustment for differences in baseline characteristics was performed using stabilized inverse probability of treatment weighting (IPTW) models. Second, we further adjusted for medication usage (aspirin, ACEI/ARB, β-blocker, statin, and oral anticoagulant). Third, we censored patients who died within 1 month after discharge. Fourth, to minimize the potential misclassification of NOAF, we repeated the analysis by excluding those with prior stroke/TIA who were at high risk of asymptomatic AF (1, 2). Moreover, we performed an exploratory analysis in which patients with or without AF symptoms were further grouped according to the NOAF burden of 10.87% to investigate its interaction effects with AF symptoms, given the prognostic importance of the burden of post-MI NOAF (17). All analyses were performed with Stata v14.0 and R v3.6.3. A two-sided *P* < 0.05 was thought to be statistically significant.

## Results

### Study Population

Among the 2,399 participants included in the NOAFCAMI-SH registry, 278 (11.6%) developed NOAF during their hospital stay. Among those, 145 (6.0%) experienced asymptomatic NOAF and 133 (5.5%) had symptomatic NOAF. Baseline characteristics are shown in Table 1. Patients in the NOAF group were older, mainly female, more likely to have a history of HF, with a higher GRACE score and CHA_2_DS_2_-VASc score, with a lower LVEF value, and less likely to undergo PCI for reperfusion when compared with those in the SR group. No significant difference was observed between asymptomatic and symptomatic NOAF except admission heart rate. Table 2 demonstrates the use of medications. Patients with symptomatic NOAF were more likely to be prescribed vasoactive agents, diuretics, and amiodarone when compared with the other two groups.

**Table 1:** Patient characteristics.

	**Sinus rhythm**	**Asymptomatic NOAF**	**Symptomatic NOAF**	***P*-value**
	**(*N* = 2,121)**	**(*N* = 145)**	**(*N* = 133)**	
**Demography and medical history**				
Age (years), mean ± SD	64.7 ± 12.2*^‡^	73.8 ± 11.2	74.8 ± 9.7	<0.001
Men	1,651 (77.8)*^‡^	96 (66.2)	90 (67.7)	<0.001
Body mass index (kg/m^2^), mean ± SD	24.6 ± 3.3	24.4 ± 3.4	24.2 ± 3.7	0.570
Current smoker	959 (45.2)*^‡^	46 (31.7)	38 (28.6)	<0.001
Hypertension	1,353 (63.8)	100 (69.0)	96 (72.2)	0.076
Diabetes mellitus	804 (37.9)	57 (39.3)	58 (43.6)	0.409
Hyperlipidemia	578 (27.3)	29 (20.0)	29 (21.8)	0.072
Chronic kidney disease	185 (8.7)	20 (13.8)	14 (10.5)	0.103
History of heart failure	105 (5.0)^*‡^	20 (13.8)	16 (12.0)	<0.001
Peripheral artery disease	75 (3.5)*	11 (7.6)	9 (6.8)	0.012
Prior AMI	139 (6.6)	11 (7.6)	15 (11.3)	0.106
Prior PCI	184 (8.7)	18 (12.4)	17 (12.8)	0.103
Prior stroke/TIA	237 (11.2)^‡^	22 (15.2)	28 (21.1)	0.001
**Initial presentation**				
Out-of-hospital cardiac arrest	39 (1.8)	6 (4.1)	6 (4.5)	0.026
STEMI	1,299 (61.2)	93 (64.1)	81 (60.9)	0.781
On admission Killip > I	291 (13.7)^*, ‡^	41 (28.3)	45 (33.8)	<0.001
SBP (mmHg), median (IQR)	137 (120–154)^‡^	136 (120–156)	130 (111–151)	0.031
HR (bpm), median (IQR)	79 (69–90)^‡^	80 (68–93)^†^	89 (72–104)	<0.001
GRACE risk score, mean ± SD	118.8 ± 28.0^*‡^	141.1 ± 28.9	149.2 ± 27.6	<0.001
CHA_2_DS_2_-VASc score, mean ± SD	2.5 ± 1.8^*‡^	3.6 ± 1.8	4.0 ± 1.8	<0.001
**In-hospital examination and outcomes**				
Creatinine (mg/dl)	0.88 (0.75–1.04)^*‡^	0.95 (0.81–1.29)	1.04 (0.85–1.37)	<0.001
Peak troponin-T (ng/ml)	2.87 (0.77–7.76)^*, ‡^	4.67 (0.80–10.00)	4.24 (1.12–9.93)	0.006
Log peak NT-pro BNP (pg/ml)	3.14 (2.83–3.50)^*‡^	3.57 (3.24–3.96)	3.82 (3.49–4.18)	<0.001
PCI with stent	1,764 (83.2)^*‡^	108 (74.5)	91 (68.4)	<0.001
Pre-PCI TIMI flow 2 or 3	1,006 (52.3)^‡^	52 (42.6)	43 (38.7)	0.003
Post-PCI TIMI flow 2 or 3	1,886 (98.0)^‡^	119 (97.5)	102 (91.9)	<0.001
Left atrial diameter (mm)	38 (35–41)^*‡^	40 (37–43)	41 (36–43)	<0.001
LVEF (%)	53 (43–60)^*‡^	50 (38–58)	45 (34–55)	<0.001
Total CEM duration (hours)	144.6 (109.0–198.3)^*‡^	185.5 (141.6–277.0)	211.4 (151.7–307.3)	<0.001
Total AF duration (hours)	–	13.4 (5.0–70.7)	14.4 (3.8–64.0)	0.518
AF burden (%)	–	8.41 (2.79–37.63)	8.41 (1.56–35.52)	0.445
Longest AF episode duration (hours)	–	11.0 (4.7–57.1)	8.9 (2.7–42.8)	0.175
AF from admission duration (hours)	–	28.9 (6.5–71.9)	29.6 (4.0–56.9)	0.187
Maximum HR in AF rhythm (bpm)	–	90 (74–114)^†^	142 (128–156)	<0.001
In-hospital death	106 (5.0)^‡^	10 (6.9)^†^	32 (24.1)	<0.001
Length of hospitalization (days)	7 (5–9)^*‡^	8 (6–12)	9 (7–13)	<0.001

Values presented as mean ± SD, median (IQR), or n (%). The level of statistical significance was p < 0.017 for *sinus rhythm vs. asymptomatic NOAF;

† *asymptomatic NOAF vs. symptomatic NOAF; and*

‡ *sinus rhythm vs. symptomatic NOAF, after multiple comparisons. AMI, acute myocardial infarction; CEM, continuous electronic monitoring; HR, heart rate; LVEF, left ventricular ejection fraction, GRACE, Global Registry of Acute Coronary Events; PCI, percutaneous coronary intervention; SBP, systolic blood pressure; TIA, transient ischemic attack; TIMI, thrombolysis in myocardial infarction*.

**Table 2:** Medications during hospitalization and at discharge.

	**Sinus rhythm**	**Asymptomatic NOAF**	**Symptomatic NOAF**	***P*-value**
	**(*N* = 2,121)**	**(*N* = 145)**	**(*N* = 133)**	
**Medications during hospitalization**				
Aspirin	2,021 (95.3)	140 (96.6)	122 (91.7)	0.130
P2Y_12_ receptor inhibitor	2,086 (98.3)	140 (96.6)	132 (99.2)	0.185
GP II_b_/III_a_ inhibitor	1,803 (85.0)	123 (84.8)	107 (80.5)	0.366
Vasoactive agent	524 (24.7)^*^^‡^	56 (38.6)^†^	75 (56.4)	<0.001
Oral anticoagulant	2 (0.1)^*^^‡^	2 (1.4)	4 (3.0)	<0.001
ACEI/ARB	1,326 (62.5)	1,326 (62.5)	86 (64.7)	0.876
β-blocker	1,626 (76.7)	100 (69.0)	103 (77.4)	0.103
Statin	2,073 (97.7)	139 (95.9)	129 (97.0)	0.328
Diuretic	665 (31.4)^*^^‡^	95 (65.5)^†^	110 (82.7)	<0.001
Amiodarone	237 (11.2)^*^^‡^	67 (46.2)^†^	118 (88.7)	<0.001
**Medications at discharge**				
Aspirin	1,858 (92.2)	119 (88.1)	88 (87.1)	0.057
P2Y_12_ receptor inhibitor	1,941 (96.3)*	122 (90.4)	98 (97.0)	0.002
Oral anticoagulant	2 (0.1)^*^^‡^	6 (4.4)	4 (4.0)	<0.001
ACEI/ARB	1,205 (59.8)	73 (54.1)	54 (53.5)	0.208
β-blocker	1,488 (73.8)*	77 (57.0)	64 (63.4)	<0.001
Statin	1,939 (96.2)*	123 (91.1)	96 (95.0)	0.014
Diuretic	258 (12.8)^*^^‡^	37 (27.4)	31 (30.7)	<0.001
Amiodarone	25 (1.2)^*^^‡^	11 (8.1)^†^	21 (20.8)	<0.001

*Values presented as n (%). The level of statistical significance was p < 0.017 for*

* *sinus rhythm vs. asymptomatic NOAF;*

† *asymptomatic NOAF vs. symptomatic NOAF; and*

‡ *sinus rhythm vs. symptomatic NOAF, after multiple comparisons. ACEI/ARB, angiotensin-converting enzyme inhibitors/angiotensin receptor blocker*.

### In-hospital Mortality

A total of 148 (6.2%) patients died during hospitalization, of whom 106 (5.0%), 10 (6.9%), and 32 (24.1%) were in the SR, asymptomatic NOAF, and symptomatic NOAF groups, respectively. As shown in Table 3, when treating the SR as the reference, the fully adjusted ORs and 95% CIs were 0.60 (0.28–1.29) and 2.35 (1.38–3.98) for the asymptomatic and symptomatic NOAF, respectively.

**Table 3:** Unadjusted and multivariable-adjusted logistic models for in-hospital mortality.

	**Unadjusted**	***P*-value**	**Model 1**	***P*-value**	**Model 2**	***P*-value**	**Model 3**	***P*-value**
	**OR (95% CI)**		**OR (95% CI)**		**OR (95% CI)**		**OR (95% CI)**	
Sinus rhythm	Reference	–	Reference	–	Reference	–	Reference	–
Asymptomatic NOAF	1.41 (0.72–2.76)	0.318	0.79 (0.39–1.57)	0.500	0.85 (0.42–1.70)	0.642	0.53 (0.24–1.17)	0.117
Symptomatic NOAF	6.02 (3.87–9.38	<0.001	3.59 (2.26–5.70)	<0.001	3.59 (2.24–5.74)	<0.001	2.32 (1.36–3.94)	0.002

*Model 1 is adjusted for age and sex. Model 2 is adjusted for model 1 + cardiovascular risk factors (current smoker, hypertension, diabetes, prior stroke, prior PAD, and prior MI). Model 3 is adjusted for model 2 + admission characteristics (Killip class, heart rate, SBP, out-of-hospital cardiac arrest, LVEF, and creatinine) and in-hospital PCI. CI, confidence interval; OR, odds ratio; PAD, peripheral artery disease. Other variables refer to Table 1*.

### Long-Term Outcomes

**Figure 1:**
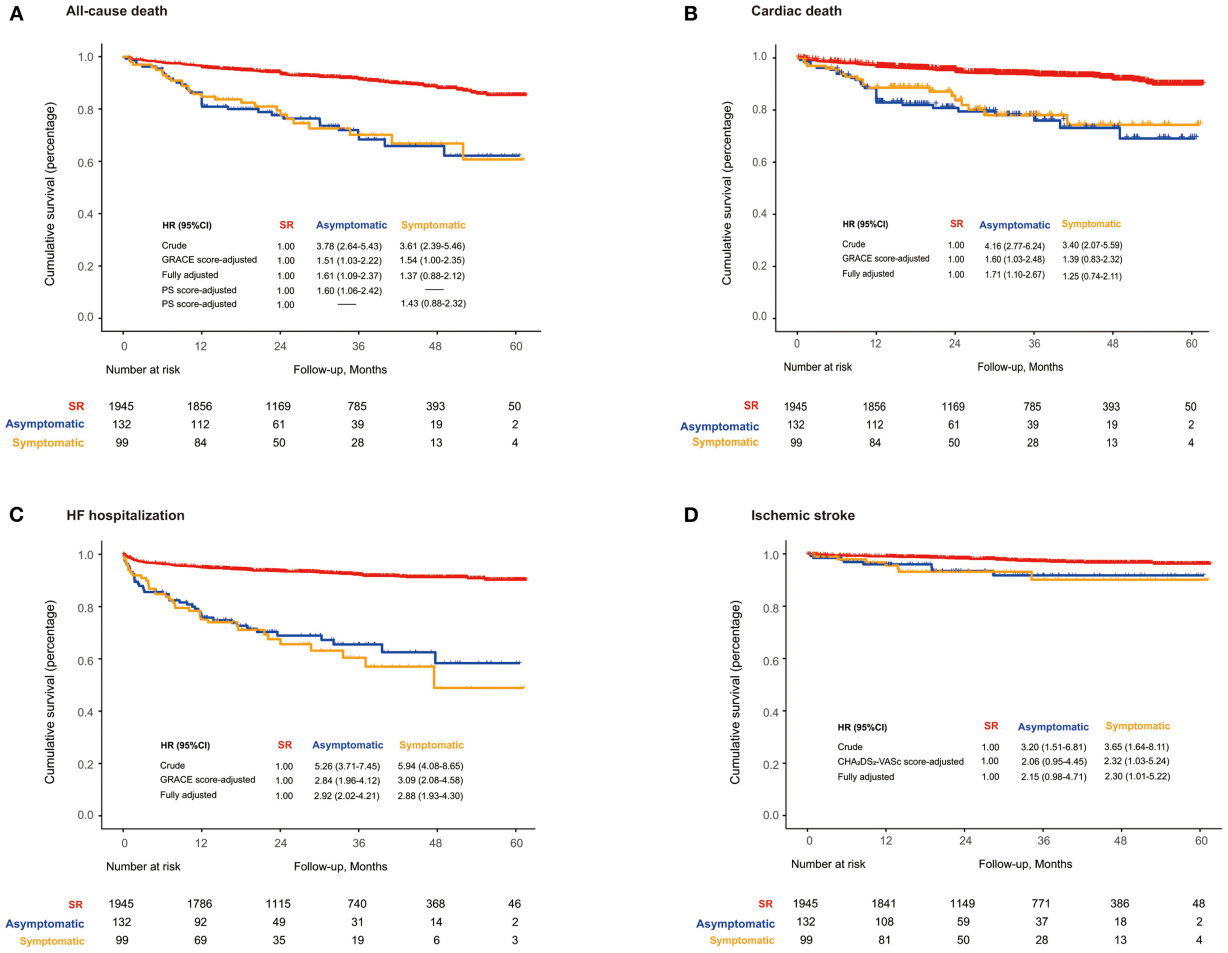
**(A)** Long-term all-cause death; **(B)** cardiovascular death; **(C)** HF hospitalization-free survival; **(D)** ischemic stroke-free survival based on the symptom of NOAF during AMI. HF, heart failure; NOAF, new-onset atrial fibrillation; PS, propensity score; SR, sinus rhythm.

Over a median follow-up of 2.7 years (IQR: 1.6–3.9), all-cause mortality was 3.2% (2.7–3.7%) for the SR, 12.4 (8.9–17.2%) for asymptomatic NOAF, and 11.8% (8.0–17.3%) for symptomatic NOAF. When compared with SR, the HRs and 95% CIs were 1.51 (1.03–2.22) for asymptomatic NOAF and 1.54 (1.00–2.35) for symptomatic NOAF after accounting for GRACE score and 1.61 (1.09–2.37) and 1.37 (0.88–2.12), respectively, after full adjustment. After adjustment for the propensity scores, the HR for asymptomatic NOAF was 1.60 (1.06–2.42) and 1.43 (0.88–2.32) for symptomatic NOAF, compared with the SR (Figure 1A). Moreover, it was the asymptomatic NOAF (HR: 1.71, 95% CI: 1.10–2.67) rather than the symptomatic one (HR: 1.25, 95% CI: 0.74–2.11) that was significantly associated with elevated cardiovascular mortality (Figure 1B). Both asymptomatic (HR: 2.92, 95% CI: 2.02–4.21) and symptomatic NOAF (HR: 2.88, 95% CI: 1.93–4.30) episodes were significantly associated with increased risk of HF hospitalization (Figure 1C). Only patients with symptomatic NOAF were at heightened long-term risk of ischemic stroke compared to those with SR (HR: 2.30, 95% CI: 1.01–5.22; Figure 1D).

**Figure 2:**
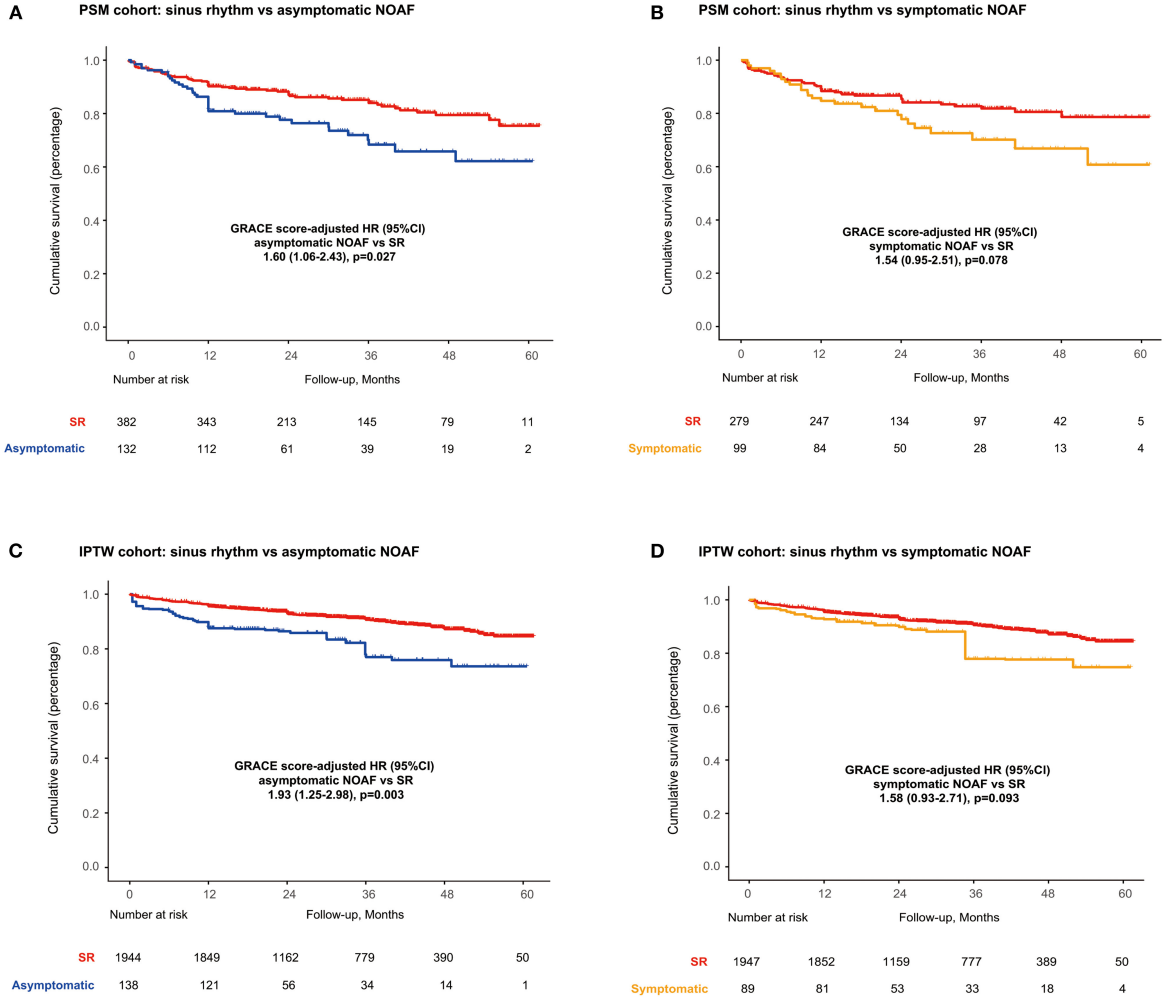
Long-term survival in PSM cohorts for **(A)** SR vs. asymptomatic NOAF and **(B)** SR vs. symptomatic NOAF and in IPTW cohorts for **(C)** SR vs. asymptomatic NOAF and **(D)** SR vs. symptomatic NOAF. IPTW, inverse probability of treatment weighting; PSM, propensity score matching.

As illustrated in Supplementary Figure 2, patient's characteristics were well-balanced in the propensity score-matched (PSM) cohorts. In the matched cohorts, long-term mortality was 6.3% (4.9–8.1%) for the SR and 12.4% (8.9–17.2%) for asymptomatic NOAF (asymptomatic NOAF vs. SR, HR: 1.60, 95% CI: 1.06–2.43; *P* = 0.027) and 7.0% (5.2–9.4%) for the SR and 11.8% (8.0–17.3%) for symptomatic NOAF (symptomatic NOAF vs. SR, HR: 1.58, 95% CI: 0.93–2.71; *P* = 0.093) (Figures 2A,B). IPTW analyses demonstrated similar results (Figures 2C,D).

**Figure 3:**
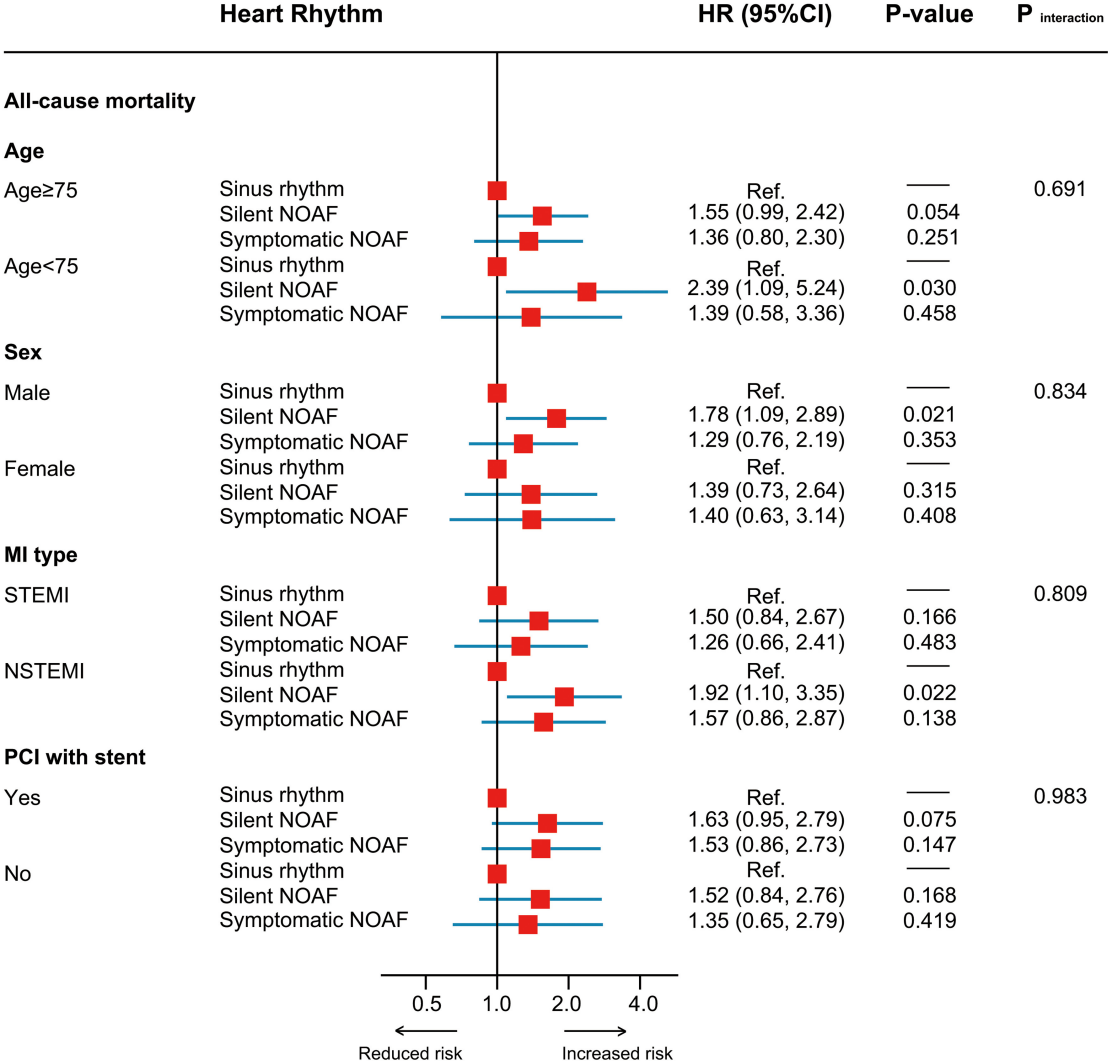
Subgroup analysis. MI, myocardial infarction; PCI, percutaneous coronary intervention.

**Figure 4:**
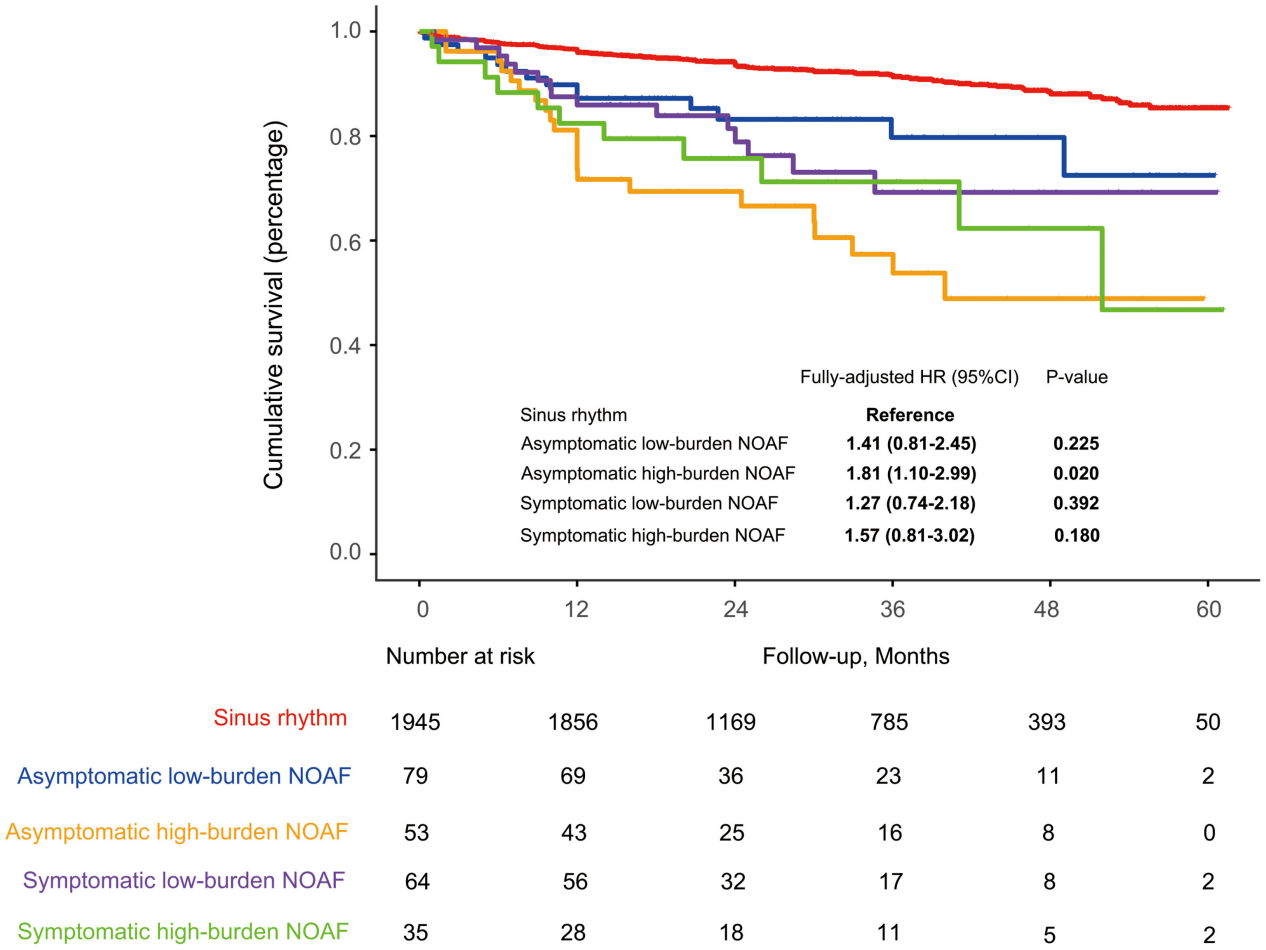
Long-term survival in the whole cohort stratified by the symptom and burden level of NOAF during AMI. HF, heart failure; NOAF, new-onset atrial fibrillation; PS, propensity score; SR, sinus rhythm.

No significant heterogeneity in HR was observed across all subgroups (Figure 3). In the sensitivity analysis, results remained robust after adjustment for the medication usage, censoring patients who died within 1 month after discharge or excluding those with a prior stroke/TIA (Supplementary Figure 3). In the exploratory analysis, patients with asymptomatic high-burden NOAF were identified as the highest-risk population with all-cause mortality of 19.6% per year [fully adjusted HR (treating SR as the reference): 1.81, 95% CI: 1.10–2.99, *P* = 0.020; Figure 4).

## Discussion

The present analysis found that the incidence rate of post-MI asymptomatic NOAF was 6.0%. Symptomatic NOAF episodes were significantly associated with increased in-hospital mortality, whereas only asymptomatic episodes were related to poor long-term survival. Similar results were observed for the risk evaluation of cardiovascular death. Notably, patients with high-burden asymptomatic NOAF represented the highest-risk population for all-cause death. In addition, when compared to the SR, both asymptomatic and symptomatic NOAF were associated with a heightened risk of HF hospitalization, but only symptomatic NOAF was challenged by a higher risk of ischemic stroke.

AF represents the most common arrhythmia in daily clinical practice, but its incidence is still thought to be underestimated since AF sometimes can be completely asymptomatic. Nowadays, technical advances in cardiac implantable electronic devices allow for the early detection of asymptomatic AF, and the prevalence of asymptomatic AF is varied across different settings (18), but data about asymptomatic AF in the AMI population remain limited. In our study, the incidence rate of in-hospital asymptomatic NOAF was ≈6.0%, which is lower than that reported in a French registry where nearly 16.0% of the AMI individuals presented with asymptomatic AF (13). We postulated the difference might be explained as follows: the former study included patients with pre-existing AF who might have received β-blockers for rate control, thus alleviating the symptoms of AF. Besides, the high usage rate of amiodarone for cardioversion purposes reflects our concerns on the detrimental impact of post-MI NOAF, and this could also contribute to the low rate of asymptomatic AF (11, 12).

An important finding of our study was that the impacts of asymptomatic and symptomatic NOAF episodes on short- and long-term survival were divergent. After adjustment for conventional cardiovascular confounders, we showed that symptomatic NOAF was associated with 2-fold increased mortality during hospitalization; in contrast, only the asymptomatic NOAF was significantly related to poor long-term survival (HR: 1.72, 95% CI: 1.17–2.53, *P* = 0.005). The robustness of our results was further validated in the PSM and IPTW cohorts. Preceding studies had identified several pivotal risk factors of in-hospital death, such as age, heart rate, SBP, Killip class, etc. (19, 20). Given the fact that the majority of aforementioned risk factors presented in the symptomatic NOAF group (Table 1), it was not hard to understand such increased in-hospital mortality.

Debates concerning the prognostic implications of asymptomatic and symptomatic AF still exist in various settings (4, 6, 9). In a subanalysis of the Atrial Fibrillation Follow-up Investigation of Rhythm Management (AFFIRM) trial, although the crude mortality was higher in the symptomatic AF group compared with that in the asymptomatic group (27 vs. 19%), statistical significance was not achieved after adjusting for a history of coronary artery disease, HF, and LVEF (HR: 1.07, 95% CI: 0.79–1.46, *P* = 0.67) (7). By contrast, Boriani et al. demonstrated that asymptomatic AF was significantly associated with increased 1-year mortality as compared with symptomatic AF (9.4 vs. 4.2%, *P* < 0.0001) (3). This time, we demonstrated that the asymptomatic NOAF was significantly associated with poor long-term survival. Similarly, Stamboul et al. had also reported that when treating patients with SR as the reference, those with asymptomatic AF during AMI were at higher risk of 1-year cardiovascular events even after multivariate adjustment (OR: 2.24, 95% CI: 1.02–4.93, *P* = 0.046) (15). Although the exact mechanism was still unclear, we considered this could partially be ascribed to the insufficient clinical concerns for patients without AF symptoms, thus leading to the inappropriate or delayed use of optimal management (4). Besides, Guenancia et al. showed that AF recurrences were more frequent in patients with symptomatic AF during AMI than in those with asymptomatic AF, which would make the asymptomatic NOAF even more difficult to be detected and treated (21). Our exploratory analysis in which patients with asymptomatic high-burden AF episodes (AF burden >10.87%) were recognized as the highest-risk population underscored the clinical importance of strengthened ECG monitoring and AF burden control, since the asymptomatic AF had been determined by Potpara et al. as more likely to progress into a permanent pattern when compared to the symptomatic one (HR: 1.6, 95% CI: 1.1–2.2, *P* = 0.009) (8). Technical advances with respect to AF detection, for example, the use of smart device-based photoplethysmography technology (22), may be useful in patients with NOAF during AMI for the long-term AF screening, AF burden evaluation, as well as further clinical decision-making.

In line with prior studies, our results with respect to HF hospitalization further corroborated the fact that NOAF was an important risk factor of HF after AMI (17, 23), which was independent of AF symptoms. Interestingly, we found that only the symptomatic NOAF during AMI was significantly associated with an increased risk of ischemic stroke, which was different from previous reports that patients with asymptomatic AF were at high risk of ischemic stroke due to suboptimal anticoagulation (8, 24). We assumed it might be explained by the low usage rate of oral anticoagulant (OAC) among post-MI NOAF individuals (≈7.4%); therefore, OAC treatment would have little impact on the analyzed population. In fact, as reported in the Chinese Acute Myocardial Infarction (CAMI) registry, only 5.1% of AMI patients concomitant with AF had been prescribed warfarin, and the rate of the combined use of warfarin and dual antiplatelet was even lower (≈1.7%) (25). Such a striking gap may be due to the careful prescription of OAC after AMI given the risk of intracranial hemorrhage is higher in the Asian population (26). Accordingly, based on the present study, we postulated that patients' clinical profiles could be the dominant factor for the elevated risk of ischemic stroke, as patients with symptomatic NOAF had a relatively higher CHA_2_DS_2_-VASc score compared to those with asymptomatic NOAF (3.6 ± 1.8 vs. 4.0 ± 1.8; Table 1).

### Limitations

The present analysis is retrospective in nature and thus subject to limitations about the uniformity of data collection. However, we performed a manual review of all admission records, rather than rely on coded information to both adjudicate the diagnosis of AMI as well as NOAF ascertainment. Although patients with a documented history of AF had been excluded, we cannot eliminate the possibility of NOAF misclassification as patients with an undiagnosed AF may be included. Because of lacking data on the specific causes of death (e.g., due to HF, stroke, bleeding, etc.), we cannot evaluate the association of the NOAF symptoms with cause-specific mortality. Also, the low rate of oral anticoagulant usage may limit the generalization of our results to other cohorts. Finally, the number of patients who developed NOAF in this study is limited, and further studies with a larger sample size are highly desirable to confirm our findings.

## Conclusions

Our results indicated that patients with post-MI symptomatic NOAF were the high-risk population of in-hospital death, and those with asymptomatic NOAF, especially concomitant with a high AF burden, had poor long-term survival. These findings highlight the importance of strengthened management for symptomatic NOAF episodes during the acute phase of AMI and the usefulness of extensive ECG monitoring among patients with asymptomatic NOAF to facilitate AF detection as well as timely initiation of treatment.

## Data Availability Statement

The raw data supporting the conclusions of this article will be made available by the authors, without undue reservation.

## Ethics Statement

The studies involving human participants were reviewed and approved by The ethics committee of the Shanghai Tenth People's Hospital. The ethics committee waived the requirement of written informed consent for participation.

## Author Contributions

All authors listed have made a substantial, direct and intellectual contribution to the work, and approved it for publication.

## Funding

This work was supported by the Natural Science Foundation of Shanghai (18ZR1429700) and the National Natural Science Foundation of China (81270193 and 81900385).

## Conflict of Interest

The authors declare that the research was conducted in the absence of any commercial or financial relationships that could be construed as a potential conflict of interest.

## Publisher's Note

All claims expressed in this article are solely those of the authors and do not necessarily represent those of their affiliated organizations, or those of the publisher, the editors and the reviewers. Any product that may be evaluated in this article, or claim that may be made by its manufacturer, is not guaranteed or endorsed by the publisher.
