# Supplementary material for: Prognostic Impact of the Symptom of New-Onset Atrial Fibrillation in Acute Myocardial Infarction: Insights From the NOAFCAMI-SH Registry
Source: Front Cardiovasc Med. 2021 Sep 22;8:677695. doi: 10.3389/fcvm.2021.677695 (PMC8492948; doi:10.3389/fcvm.2021.677695)
Supplement: Supplementary file 1 [file Data_Sheet_1.docx]

**SUPPLEMENTAL MATERIAL**

1. Supplementary Methods

2. Online Figure 1. Flow diagram of the present analysis

3. Online Figure 2. Baseline characteristics comparison before and after propensity-score matching analysis

4. Online Figure 3. Sensitivity analysis

**Supplementary Methods**

In our propensity-score matching analysis, the covariates used to calculate the propensity scores included: age, sex, current smoker, a history of hypertension, diabetes, dyslipidemia, chronic kidney disease, heart failure, stroke/transient ischemic attack, myocardial infarction, percutaneous coronary intervention and peripheral artery disease, STEMI, on-admission systolic blood pressure, heart rate, and heart failure (Killip>I), and out-of-hospital cardiac arrest.


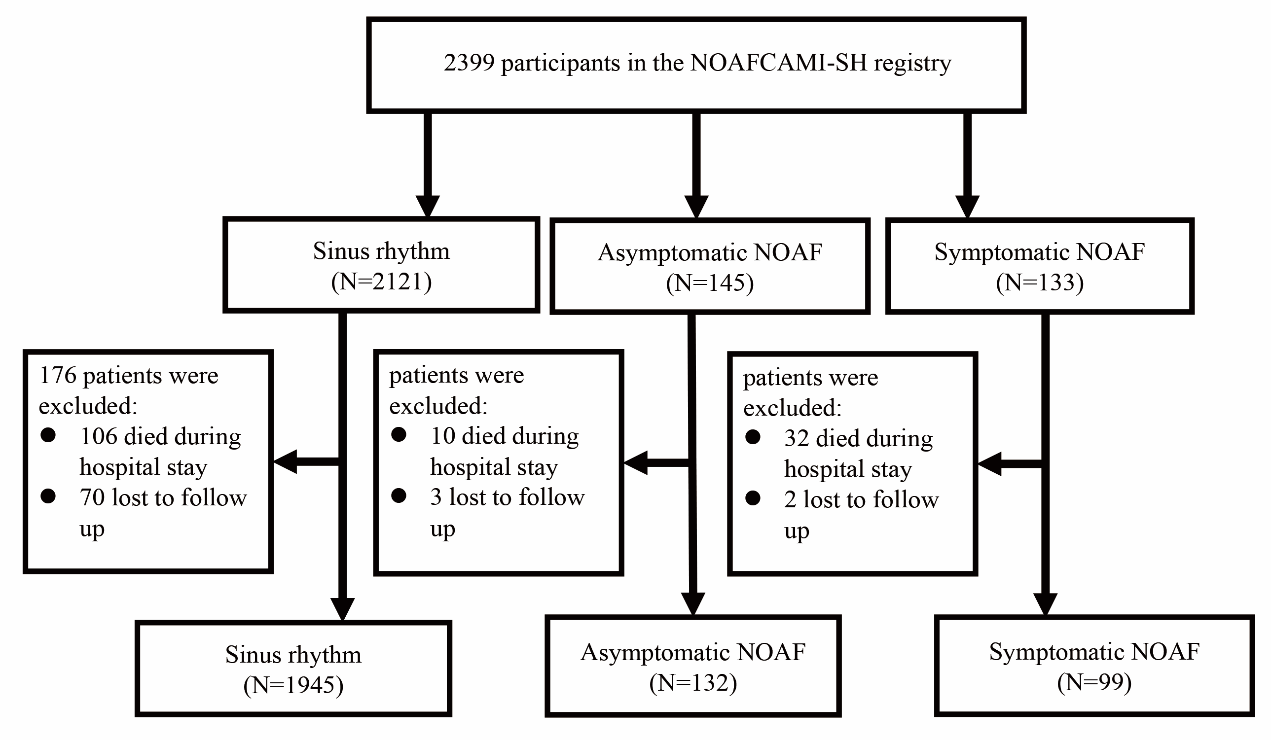


Online Figure 1. Flow diagram of the present analysis


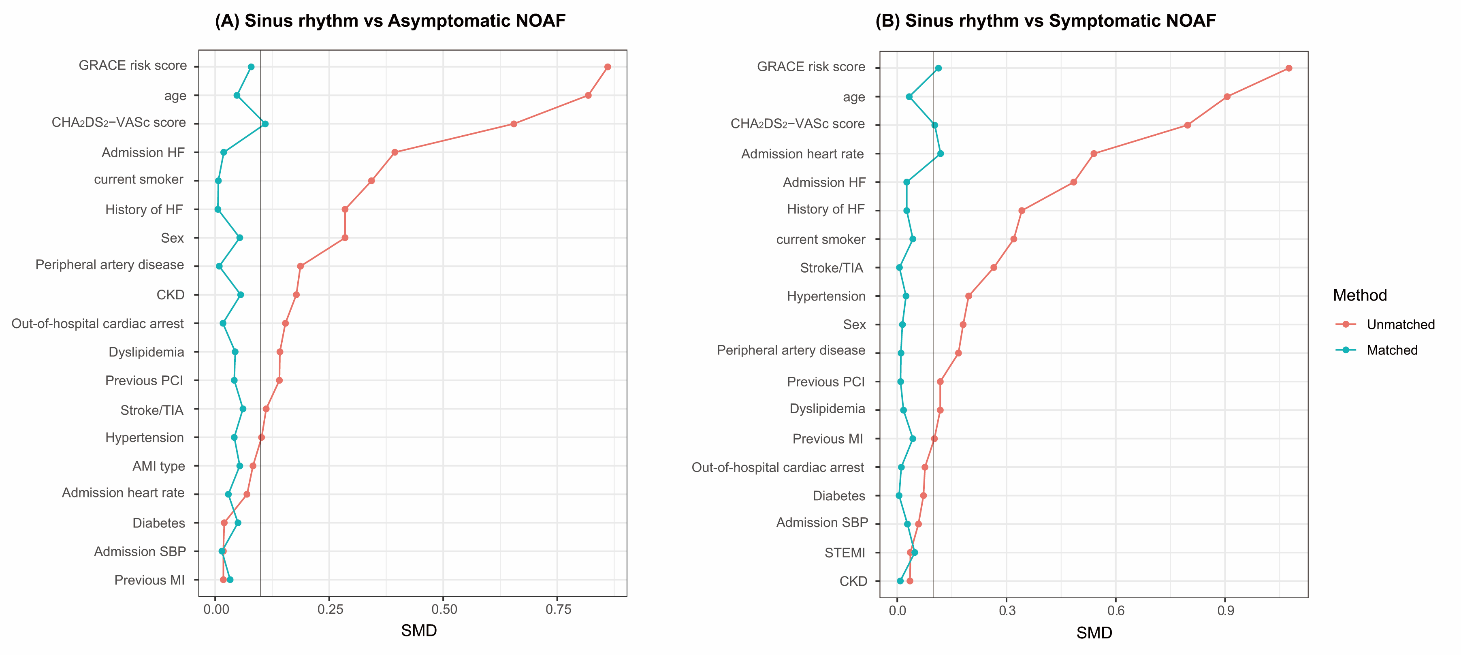


Online Figure 2. Baseline characteristics comparison before and after propensity-score matching analysis


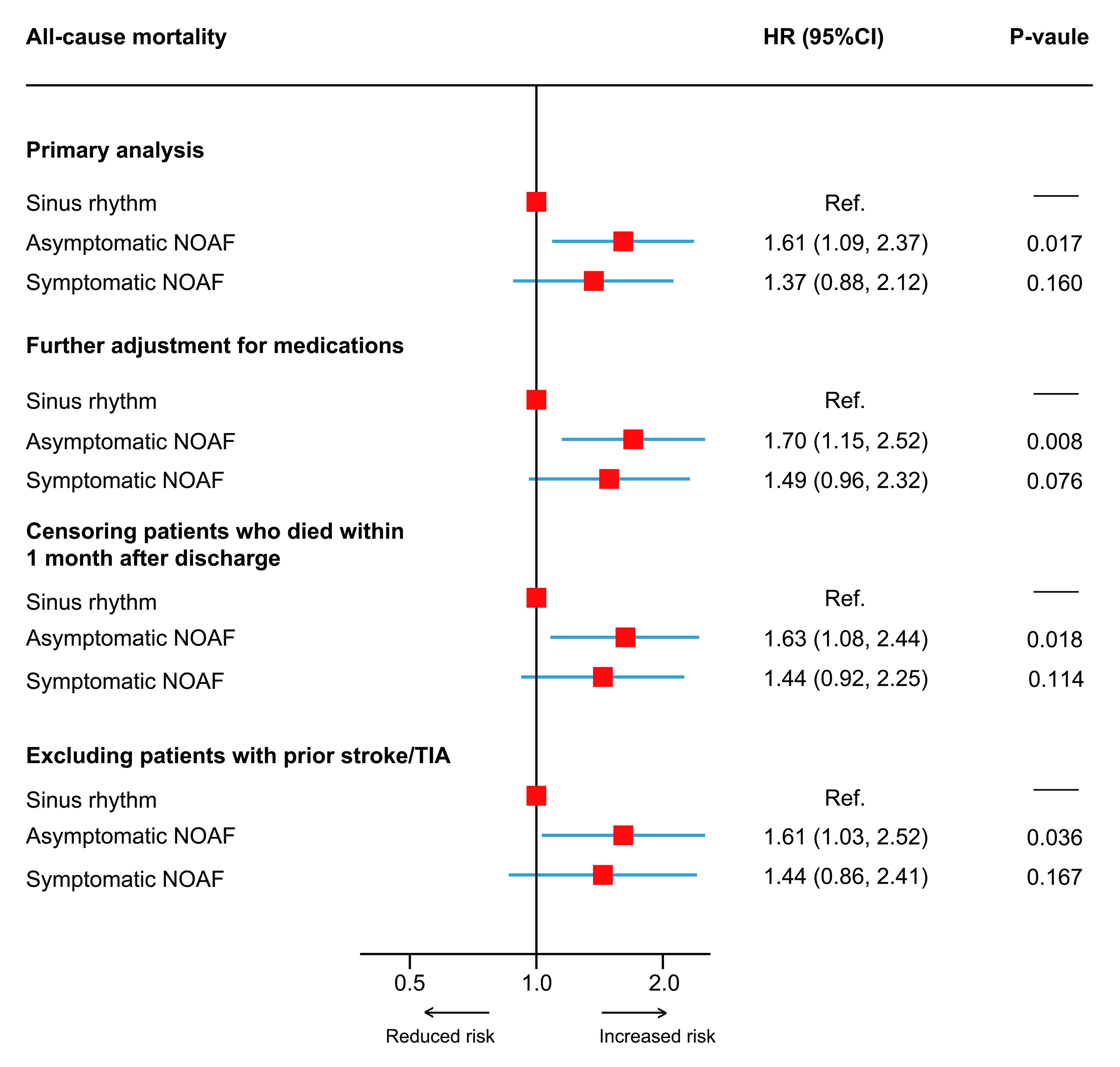


Online Figure 3. Sensitivity analysis
